# Predicting performance using background characteristics of international medical graduates in an inner-city university-affiliated Internal Medicine residency training program

**DOI:** 10.1186/1472-6920-9-42

**Published:** 2009-07-13

**Authors:** Balavenkatesh Kanna, Ying Gu, Jane Akhuetie, Vihren Dimitrov

**Affiliations:** 1 Department of Internal Medicine, Lincoln Medical & Mental Health Center, New York, USA; 2 Weill Medical College of Cornell University, Ithaca, NY, USA; 3 Department of Internal Medicine, Lincoln Medical & Mental Health Center, New York, USA

## Abstract

**Background:**

IMGs constitute about a third of the United States (US) internal medicine graduates. US residency training programs face challenges in selection of IMGs with varied background features. However data on this topic is limited. We analyzed whether any pre-selection characteristics of IMG residents in our internal medicine program are associated with selected outcomes, namely competency based evaluation, examination performance and success in acquiring fellowship positions after graduation.

**Methods:**

We conducted a retrospective study of 51 IMGs at our ACGME accredited teaching institution between 2004 and 2007. Background resident features namely age, gender, self-reported ethnicity, time between medical school graduation to residency (pre-hire time), USMLE step I & II clinical skills scores, pre-GME clinical experience, US externship and interest in pursuing fellowship after graduation expressed in their personal statements were noted. Data on competency-based evaluations, in-service exam scores, research presentation and publications, fellowship pursuance were collected. There were no fellowships offered in our hospital in this study period. Background features were compared between resident groups according to following outcomes: (a) annual aggregate graduate PGY-level specific competency-based evaluation (CBE) score above versus below the median score within our program (scoring scale of 1 – 10), (b) US graduate PGY-level specific resident in-training exam (ITE) score higher versus lower than the median score, and (c) those who succeeded to secure a fellowship within the study period. Using appropriate statistical tests & adjusted regression analysis, odds ratio with 95% confidence intervals were calculated.

**Results:**

94% of the study sample were IMGs; median age was 35 years (Inter-Quartile range 25th – 75th percentile (IQR): 33–37 years); 43% women and 59% were Asian physicians. The median pre-hire time was 5 years (IQR: 4–7 years) and USMLE step I & step II clinical skills scores were 85 (IQR: 80–88) & 82 (IQR: 79–87) respectively. The median aggregate CBE scores during training were: PG1 5.8 (IQR: 5.6–6.3); PG2 6.3 (IQR 6–6.8) & PG3 6.7 (IQR: 6.7 – 7.1). 25% of our residents scored consistently above US national median ITE scores in all 3 years of training and 16% pursued a fellowship.

Younger residents had higher aggregate annual CBE score than the program median (p < 0.05). Higher USMLE scores were associated with higher than US median ITE scores, reflecting exam-taking skills. Success in acquiring a fellowship was associated with consistent fellowship interest (p < 0.05) and research publications or presentations (p <0.05). None of the other characteristics including visa status were associated with the outcomes.

**Conclusion:**

Background IMG features namely, age and USMLE scores predict performance evaluation and in-training examination scores during residency training. In addition enhanced research activities during residency training could facilitate fellowship goals among interested IMGs.

## Background

The numbers of international medical graduates (IMGs) who train in the United States (US) have been steadily increasing over the past three decades [1]. IMGs continue to account for a growing proportion of the internal medicine generalists and subspecialists [2]. Forty four percent of residents in internal medicine programs in the United States are immigrant physicians [3]. Residency programs are challenged in assisting IMGs with different backgrounds to fit the US healthcare system and accomplish their career goals. In order to facilitate this process, programs require guidance in selection of IMGs with varied backgrounds for graduate medical education (GME) training in the US. We found very little published data in this topic. A 1990 survey of 102 directors of internal medical residencies with the largest numbers of foreign-born foreign medical graduates revealed that the most important performance predictors were scores on the foreign medical graduate examination or national board of medical examiners test, interviews, and U.S. postgraduate clinical experience. [4] Also, another old study showed that clinical experience and performance on standardized examinations are the two selection criteria most predictive of foreign-born IMGs' first-year performances as internal medicine residents. [5] There are no recent studies on this topic especially when the demographic features, clinical experience and career choices of IMGs are changing constantly over time. [6]

This study was designed to explore the characteristics of residents of an internal medicine training program with IMGs from varied backgrounds and the association of these characteristics to clinical performance, examination performance and career choice in the US.

## Methods

We conducted a retrospective, observational case-control study of 51 consecutive internal medicine residents who had successfully completed training in internal medicine at Lincoln Medical and Mental Health Center (LMMHC) between 2004 and 2007. LMMHC is an inner-city university-affiliated institution with an American college of graduate medical education (ACGME) accredited internal medicine three-year training program consisting of 84 residents.

The study was reviewed and approved by the local institutional review board. Two trained independent reviewers who were not associated with the internal medicine department and blinded to study protocol extracted data on study subjects from the graduate medical education (GME) records to limit bias and protect confidentiality of data.

Data was collected on selected background independent variables, including resident age, gender, nationality, medical school graduation year, United States medical licensing examination (USMLE) scores, pre-GME clinical experience, especially externship training in the United States, pre-GME research experience, awards, and fellowship aspirations documented in participant's personal statements. In addition, information on the dependent variables namely, ACGME competency based monthly formative CBE scores, detailed internal medicine ITE scores, GME research activities such as publications and or presentations was also collected. Resident records without complete information on any of the above variables were excluded from review.

We measured resident performance based on three important outcome criteria as follows:

### i) Evaluation of Resident's clinical performance

#### a) PG level specific comparative formative competency-based evaluation score

The ACGME competency based PG level specific formative CBE is scored on a scale of 1 (worst) to 10 (best). These evaluations rated by supervising faculty during all 33 rotations during the 3 year period of training were extracted from the records of all participants. The annual aggregate CBE score was calculated for participants at every PG level of training. A median aggregate CBE score for each PG level was then developed utilizing the annual aggregate resident individual CBE scores. Residents were categorized based on the rating below or above PG level specific median score during all three years of training.

### ii) Evaluation of Resident examination performance

Our program's American Board of Internal Medicine (ABIM) pass-rate was 96% during the study period. Further, ABIM examination scores of all residents are not available to the individual programs. However, the ITE examinations have been validated as reflecting performance of residents on the ABIM examinations. [7] Therefore, we utilized the readily available ITE scores to assess the exam taking skills of the study participants.

#### a) ITE Total score

The percentile scores of each resident compared to the US median scores were noted. Residents were categorized as either above or below US national median scores.

#### b) General internal medicine (GIM) Score

The percentile scores of each resident in GIM section of the ITE were noted and classified as above or below median US national GIM ITE scores.

#### c) Subspecialty (SS) Score

The percentile scores of each resident in subspecialty sections of the ITE were noted and classified as above or below median US national subspecialty ITE scores.

### iii) Evaluation of pursuit of fellowship opportunity

LMMHC does not offer free-standing fellowship programs similar to certain other US Programs. [9] However, fellowship positions are desired by residents. Therefore, we also analyzed the background characteristics of study participants who pursued a fellowship position of their choice within three years of graduation.

We chose the outcome of clinical performance to assess ACGME competency based evaluation scores that are widely used in resident evaluations, for promoting them and also for their graduation. The ITE scores were measured to compare our program participants against the national performance and therefore provide generalizability of our findings in terms of examination performance. Lastly, fellowship pursuance was chosen as an outcome, assuming a higher level of difficulty in obtaining a fellowship for residents from programs without fellowship programs.

The primary aim of the study is to analyze the background characteristics of residents and the association to program evaluations, ITE scores and fellowship pursuance as described above. The study groups were divided and compared as follows: a) residents who were evaluated higher than PG level median of residents in our program in all three years of training to those who were evaluated lower, b) residents whose ITE scores were above median national scores versus those with ITE scores below the national median scores, and c) residents who pursued fellowship opportunities within three years of graduation to those who did not choose or pursue a fellowship.

Statistical analyses were performed using non-parametric Fischer's exact test and Mann Whitney U test for categorical and continuous variables respectively. Stratified analysis was performed when confounder variables were encountered. A p value of < 0.05 was considered significant.

In order to limit observer bias as well as maintain the confidentiality of records, the resident investigator was not involved in gathering data from individual resident files. The author who analyzed the data was not involved in data collection.

## Results

### Background characteristics

A total of 51 IMG residents from 22 different countries were included in the study. Twenty one other residents were excluded due to lack of data on one or many of the study variables. Baseline characteristics are described in detail in Table 1.

**Table 1:** Resident background characteristics

**Characteristics**	**Study aggregate**
Age (year), Median (IQR*)	35 (33–37)
Gender (W:M)	43%/57%
Married	65%
Time from medical school to residency training (years), Median (IQR*)	5 (4–7)
Visa status (J1/H1/Employment authorization)	88%
Asian origin	59%
USMLE Step 1, Median (IQR*)	85 (80–88)
USMLE Step 2 Clinical skills, Median (IQR*)	82 (79–87)
Pre-GME Clinical experience, n (%)	43/51 (84)
Pre-GME Research Experience, n (%)	34/51 (66.7)
US Externship, n (%)	14/51 (27.5)
Pre-GME Awards, n (%)	26/51(50.1)
ABIM examination pass, n (%)	49/51(96)
Fellowship aspirants, n (%)	12/51 (23.5)
Fellowship within 3 years after graduation, n (%)	8/51 (16)

* IQR: Inter-Quartile Range 25^th ^– 75^th ^Percentile

### Evaluation of Resident Clinical Performance

The median clinical performance annual aggregate CBE scores are 5.8 (PG 1 level); 6.3 (PG level 2) and 6.7 (PG level 3). 23 PG 1 level residents (45%), 25 PG 2 level (49%) and 25 PG 3 level (49%) residents were noted to be above the median annual aggregate CBE score of their respective PG level. Eighteen (35%) residents were consistently above the annual aggregate median CBE score during all 3 years of training.

Compared to residents whose clinical performance was consistently below median evaluation scores, residents who scored better were of younger median age (33 versus 36 years, p < 0.05)and had higher median USMLE step II clinical skills scores (86 versus 80, p < 0.05). Gender, marital status, nationality, time from medical school to residency training, temporary visa status, USMLE step I score, pre-GME clinical or research experience, and US externship were not significantly associated (p = NS) with resident clinical performance related CBE scores. (Table 2)

**Table 2:** Background characteristics and Evaluation of Clinical Performance

**Characteristics**	**Above PG level median (n = 18)**	**Below PG level median (n = 33)**	*P value
Age, median (range) in years	33 (31–40)	36 (31–47)	**< 0.05**

Male:Female (ratio)	9:9	20:13	NS

Marital Status (unmarried: married)	5:13	13:20	NS

Asian/Others	3/5	16/17	NS

#USMLE step I median (range)	86 (76–97)	83 (75–96)	NS
USMLE step II Clinical skills median (range)	86 (77–93)	80 (75–92)	**< 0.05**

Time from medical school to residency, median (range) in years	4 (1 – 14)	5 (1–17)	NS

Temporary Visa status (n)	16	29	NS

Pre-GME Clinical Experience (n)	15	28	NS

Pre-GME Research Experience (n)	12	22	NS

US Externship	7	7	NS

Pre-GME Awards	9	17	NS

# USMLE scores were not available for 1 participant

* Statistical significance by Mann Whitney U test for continuous variables and Fischer's exact test for categorical variables

### Evaluation of Resident In-training Examination performance

21 PG 1 level residents (41%), 18 PG 2 level (35%) and 24 PG 3 level (47%) residents were noted to be above the US national median score of the respective PG levels in the ITE. Thirteen (25.5%) residents were consistently above the PG level-specific US national median ITE score during all 3 years of training.

Compared to residents who scored lower than the PG level-specific US national median total ITE score, those who scored consistently better had higher median USMLE step I (86 versus 83, p < 0.005) and median USMLE step II clinical skills scores (86 versus 80, p < 0.05). Male residents were noted to have higher than median ITE scores than women (11/29 (37.9%) versus 2/22 (9.1%), p < 0.05). However, when stratified based on gender, men were noted to have better ITE scores if they had higher median USMLE Step I (90 versus 84, p = 0.06) and USMLE Step II clinical skills scores (89 versus 80, P < 0.05). Age, marital status, time from medical school to residency, nationality, temporary visa status, pre-GME clinical experience, pre-GME research experience, GME research, US externship were not associated with total ITE scores (Table 3).

**Table 3:** Background characteristics and National ITE scores(Total)

**Characteristics**	**Above US Median (n = 13)**	**Below US Median (n = 38)**	*P value
Age, median (range) in years	35 (32–42)	35.5 (31–47)	NS

**Male/Female (ratio)	11:2	18: 20	**< 0.05**

Marital Status (unmarried/married)	8: 5	25: 13	NS

Asian/Others	9/4	21/17	NS

#USMLE step I median (range)	86 (76–97)	83 (75–96)	**< 0.05**
USMLE step II Clinical Skills median (range)	86 (77–93)	80 (75–92)	**< 0.05**

Time from medical school to residency, median (range) in years	4 (1 – 14)	5 (1–17)	NS

Temporary Visa status (n)	11	34	NS

Pre-GME Clinical Experience (n)	15	28	NS

Pre-GME Research Experience (n)	12	22	NS

US Externship	7	7	NS

Pre-GME Awards	9	17	NS

# USMLE scores were not available for 1 participant

* Statistical significance by Mann Whitney U test for continuous variables and Fischer's exact test for categorical variables

** Gender-stratified analysis of ITE scores and USMLE scores did not show an independent gender-specific increase in ITE scores.

When specific areas of the ITE scores were examined separately in detail, 22 of 29 men (75.8%) versus 10 of 22 women (45.5%); p < 0.05) were noted to be associated significantly with higher median general internal medicine scores. Higher median USMLE step I (86.5 versus81.5, p < 0.05) & USMLE step II clinical skills (85.5 versus 81, p < 0.05) scores were associated with higher median subspecialty scores respectively. (Table 4)

**Table 4:** Background characteristics and National ITE scores(GIM & Subspecialty)

**#Characteristics**	**Above US Median**	**Below US Median**	**P value**
**GIM ITE Scores** **(Above median n = 32; Below median n = 19)**			

Male/Female (ratio) (unadjusted analysis)	22:10	7: 12	**< 0.05**

#USMLE step I median (range)	85 (77–97)	82 (75–96)	NS
USMLE step II Clinical skills median (range)	85.5 (75–93)	81 (76–87)	NS

**Sub-specialty ITE scores** **(Above median n = 14; Below median n = 37)**			

Male/Female (ratio) (unadjusted analysis)	11: 3	18: 19	NS

#USMLE step I median (range)	86.5 (76–97)	82.5 (75–90)	**< 0.05**
USMLE step II Clinical skills median (range)	85.5 (75–93)	81 (76–87)	**< 0.05**

# All background characteristics except those shown in table here were not significantly associated with GIM or subspecialty scores (p = NS)

### Evaluation of pursuit of a fellowship opportunity

Twelve residents (23.5%) expressed initial interest in fellowship training. A total of 8 residents (15.7%) pursued fellowship opportunities during the study period. Seven of the 8 (87.5%) residents who pursued a fellowship had GME research activities namely publications and/or presentations in national or local conferences (p < 0.05). (Table 5) The other resident, who obtained a fellowship and did not have GME research, pursued a medical informatics fellowship. Age, gender, marital status, nationality, temporary visa, USMLE scores, years from graduation, pre-GME clinical experience, pre-GME research experience, US externship were not found to be associated with pursuance of fellowship training.

**Table 5:** Background characteristics and Fellowship

**Characteristics (units)**	**Fellowship pursuing residents** **(n = 8)**	**Others** **(n = 43)**	**P value**
Age, median (range) in years	33 (31 – 39)	35 (31–47)	NS

Male/Female (ratio)	4:4	25: 18	NS

Marital status (unmarried/married)	6: 2	27:16	NS

Asian/Others	4/4	26/17	NS

#USMLE step I median (range)	85 (76–97)	85 (75–96)	NS
USMLE step II Clinical skills median (range)	82.5 (80–92)	82 (75–93)	NS

Time from medical school to residency, median (range) in years	5 (3 – 14)	5 (1–17)	NS

Visa status	7	38	NS

Pre-GME Clinical Experience (n)	7	36	NS

Pre-GME Research Experience (n)	5	29	NS

US Externship (n)	1	13	NS

Pre-GME Awards (n)	5	21	NS

Fellowship aspirants (n)	7	5	**< 0.05**

GME Research (n)	7	12	**< 0.05**

## Discussion

Due to lack of recent studies on this important topic and especially with the rising number of IMGs in the US system, the current study provides a unique opportunity to evaluate predictors of clinical and examination performance among recent international medical graduates in a US internal medicine program, as well as factors associated with success in obtaining a fellowship position among international medical graduates with varied professional backgrounds. Our internal medicine program attracts predominantly IMGs from several countries throughout the world. Therefore, our results may be applicable to a significant proportion of US GME programs that are IMG-dependent. The participants of our study belong to 22 different countries from 5 continents reflecting the diverse backgrounds of their pre-GME training and therefore providing an excellent opportunity to study the varied characteristics.

The diverse origins of our study participants certainly reflect the current trends in IMG origin from the Asian & European continents. [8] Given only about 27% had observed or worked in the US medical system prior to GME, special consideration may be required for such IMGs during orientation or training. Although a majority of our IMG participants (66%) reported research experience prior to GME, given the variations in standards across the world, the level of research experience could not be compared to provide any meaningful insight.

In the analysis of resident clinical performance using CBE scores, we noted that age and USMLE Step 2 clinical skills test significantly predicted residents' clinical performance during GME. One reason could be that older IMG residents seem to have fully developed work habits during their training in other countries that are hard to change and adapt to the extreme demands and unique expectations of the US medical system. The influence of USMLE examinations on clinical performance remains unexplained, but allows one to easily speculate that better clinical knowledge facilitates improved clinical performance.

Information on ABIM board scores is subject to waiver requirements and therefore not available to the training program. Hence we analyzed ITE, a well-proven surrogate measure of ABIM examination performance. [3] USMLE scores are found to be associated with better performance on ITE during GME. As described in literature, [8] this could in part reflect the exam taking skills of the residents. It is interesting to note that the overall ABIM examination scores among all candidates including IMGs have improved in the last decade. [3]

Lastly, fellowship pursuance among aspirants is an important issue among IMGs. Our study participants were mostly headed for Internal Medicine practices after graduation, although recently published data shows that IMGs significantly choose subspecialty over Internal Medicine practice. [9] The factors predicting their success in obtaining a fellowship of choice were participation of residents in research activities and their continued interest to pursue fellowship during residency training. The number of residents pursuing fellowship has increased over the study period (2, 3 & 5 respectively in 2005, 2006 & 2007]. Interestingly, our program implemented a structured research rotation for all residents in 2005 that is published elsewhere. [10] As reported in this publication, this rotation certainly stimulates scholarly interest and an increase in research activities among the residents. This could have facilitated the pursuance of fellowship by those residents who demonstrated a continued interest in fellowship training after graduation. Visa status, in contrast to other studies [11] did not predict fellowship pursuance in our small group of residents.

Although the sample size is limited by a single-center enrollment, the study certainly provides preliminary but new evidence on predictors of resident performance among IMGs. We did not have access to ABIM scores to evaluate examination performance and therefore utilized ITE scores. But studies have clearly shown that ITE scores reflect ABIM performance. Lastly, the number of study subjects seeking fellowships was smaller as our program does not offer fellowships after Internal Medicine training. Therefore predictors of pursuing a fellowship may not fully apply to other US Internal Medicine programs with fellowship positions. Despite this, our data validates that subspecialty fellowship programs may prefer GME research experience while choosing their candidates [12]

## Conclusion

Our study shows that resident clinical performance, examination scores and fellowship pursuance among residents are associated with selected resident characteristics such as age, USMLE scores and research activities. This information may be valuable for programs to identify possible areas of focus to help IMG residents recognize their strengths and weaknesses during residency training, and target efforts to integrate them into the US health care system. Multi-center studies and larger sample size are needed to substantiate our study findings in different settings.

## Competing interests

The authors declare that they have no competing interests.

## Authors' contributions

BK - Design, analysis, manuscript preparation.YG - Data collection, analysis. JA - Data collection Manuscript. editing. VD - Design Data collection manuscript preparation

## Pre-publication history

The pre-publication history for this paper can be accessed here:


